# Body condition predicts energy stores in apex predatory sharks

**DOI:** 10.1093/conphys/cou022

**Published:** 2014-06-23

**Authors:** Austin J. Gallagher, Dominique N. Wagner, Duncan J. Irschick, Neil Hammerschlag

**Affiliations:** 1 Leonard and Jayne Abess Center for Ecosystem Science and Policy, University of Miami, PO Box 248203, Coral Gables, FL 33124, USA; 2 R.J. Dunlap Marine Conservation Program, 4600 Rickenbacker Causeway, Miami, FL 33149, USA; 3 Beneath the Waves Incorporated, 110 W. Fayette Street, Suite 900, Syracuse, NY 13202, USA; 4 Rosenstiel School of Marine and Atmospheric Science, 4600 Rickenbacker Causeway, Miami, FL 33149, USA; 5 Department of Biology, University of Massachusetts at Amherst, 221 Morrill Science Center, Amherst, MA 01003, USA; 6 Organismic and Evolutionary Biology Program, University of Massachusetts Amherst, Amherst, MA 01003, USA

**Keywords:** Condition, energy, movement, natural selection, shark, trade-offs

## Abstract

Animal condition typically reflects the accumulation of energy stores (e.g. fatty acids), which can influence an individual's decision to undertake challenging life-history events, such as migration and reproduction. Accordingly, researchers often use measures of animal body size and/or weight as an index of condition. However, values of condition, such as fatty acid levels, may not always reflect the physiological state of animals accurately. While the relationships between condition indices and energy stores have been explored in some species (e.g. birds), they have yet to be examined in top predatory fishes, which often undertake extensive and energetically expensive migrations. We used an apex predatory shark (*Galeocerdo cuvier*, the tiger shark) as a model species to evaluate the relationship between triglycerides (energy metabolite) and a metric of overall body condition. We captured, blood sampled, measured and released 28 sharks (size range 125–303 cm pre-caudal length). In the laboratory, we assayed each plasma sample for triglyceride values. We detected a positive and significant relationship between condition and triglyceride values (*P* < 0.02). This result may have conservation implications if the largest and highest-condition sharks are exploited in fisheries, because these individuals are likely to have the highest potential for successful reproduction. Our results suggest that researchers may use either plasma triglyceride values or an appropriate measure of body condition for assessing health in large sharks.

## Abstract (precis)

We measured and took blood samples from large tiger sharks in the wild to determine if their body condition (a metric of health) was related to energy stores (fatty acids). Our results revealed that body condition and fatty acids were positively and significantly correlated. This is important for understanding how large, highly mobile apex predators such as tiger sharks store and use energy required for migration and mating.

## Introduction

Animals in nature typically encounter substantial constraints on growth due to trade-offs between the ability to obtain food vs. the ability to reproduce and survive (Stearns, 1992; Roff, 2001). Balancing these trade-offs can determine how natural selection acts on life-history traits, such as age at maturity and reproductive output (Stearns, 1992; Roff, 2001). The concept of accumulating energy stores for future use is a well-documented strategy for mitigating the effects of spatial or temporal resource scarcity as well as the costs of reproduction and migration (Klaassen, 1996; Jönsson, 1997; Zera and Harsham, 2001). The strategies that animals employ to accumulate these stores have been examined in a range of animals, from beetles to polar bears (Atkinson and Ramsay, 1995; Bommarco, 1998). Most often, researchers examine an index of condition to estimate body fat stores, and indirectly, an index of animal ‘health’ (e.g. Krebs and Singleton, 1993; Jakob *et al.*, 1996; Weatherhead and Brown, 1996; Green, 2001; Schulte-Hostedde *et al.*, 2001; Bearhop *et al.*, 2004; Goymann *et al.*, 2010). Condition metrics come in many different forms, but are generally measured as some relationship between body mass and body length (Jakob *et al.*, 1996; Green, 2001). However, values of condition may not always accurately reflect the physiological state of animals, and there is a need to determine whether values of condition accurately reflect more direct measures of health, such as fatty acid levels.

Establishing these relationships is especially important for animals that migrate over long distances, which is known to be energetically exhausting for many animals (Tucker, 1971; Klaassen, 1996) and for longer-lived species, in which the costs of reproduction are generally higher due to longer gestation and more developed young (e.g. Doughty and Shine, 1998). Fats are a major source of fuel for animals during long periods of metabolic demand. Three primary plasma lipids used in fuel transport are free fatty acids, cholesterol and triglycerides, the last of which has been documented as a primary energy store crucial for fuelling migrations in many taxa, such as bird species (Blem, 1976; Schaub *et al.*, 2001). Triglycerides are high-energy molecules, which consist of a glycerol molecule connected to three fatty acid chains. Triglycerides typically accumulate from the diet and are stored in the fat vacuoles of adipose cells in vertebrates. Plasma triglycerides mostly originate directly or via synthesis in the liver, and their presence in the plasma indicates lipid transport to surrounding tissues (i.e. liver and peripheral tissues), with high triglyceride values suggesting a high rate of fat deposition and utilization, although the transit of these products is likely to change with nutritional state (Robinson, 1970). Furthermore, the nutritional state of individual animals is important for events such as extensive migrations, and ultimately, to enable animals to survive and reproduce (Krebs and Singleton, 1993; Goymann *et al.*, 2010).

For several reasons, sharks represent a good system in which to test the relationship between condition and metrics of physiological state. Many shark species are threatened and undergoing population declines due to synergistic anthropogenic impacts (e.g. Gallagher *et al.*, 2012), and therefore, there is substantial interest in their overall health and population demographics. Moreover, many large sharks undergo extensive ocean-wide migrations over thousands of kilometres, which are related to foraging and/or reproduction (Hammerschlag *et al.*, 2011; Domeier and Nasby-Lucas, 2013). Finally, most sharks exhibit long gestation periods, have few offspring and exhibit long periods between reproductive events, which are likely to be linked to the high energetic requirements for fuelling their developing precocious embryos (Lucifora *et al.*, 2002). Taken together, these factors indicate a need to understand the relationship between condition and physiological state and suggest that energy stores are crucial in the movement, reproduction and survival among shark species.

Sharks generally store fat in the large bi-lobed liver, which is believed to play a significant role in lipid metabolism during migrations and throughout gestation (Oguri, 1990; Watson and Dickson, 2001; Hussey *et al.*, 2009; Del Raye *et al.*, 2013). However, to our knowledge, there are no published studies investigating plasma lipid reserves in live large sharks and how these energy stores change with size or body condition, despite their documented importance in catalysing or sustaining movement and reproduction in many vertebrate species. We set out to determine the relationship between a metric of condition and an independent measure of physiological state (plasma lipid metabolites) in a wide-ranging apex predator (the tiger shark, *Galeocerdo cuvier*). Like other sharks, tiger sharks have long gestation periods (Whitney and Crow, 2007) and are known to migrate long distances (>3000 km) between the Caribbean and the mid-Atlantic (Hammerschlag *et al.*, 2012). Recently, a metric of condition was developed for large sharks that uses measurements of overall body shape (Irschick and Hammerschlag, 2014). We hypothesized that we would find a positive relationship between this metric of condition (described below) and levels of plasma lipid metabolites. We make this prediction on the premise that a larger, bulkier (i.e. ‘fatter’) shark should possess relatively larger fat stores in the liver, and therefore, higher levels of circulating plasma lipid metabolites, a prediction which has some support in other analyses (Lambert and Dutil, 1997). An alternative possibility (i.e. the null hypothesis) is that there is no relationship between these variables, and that body girth is either not a good metric of body condition and/or the levels of plasma lipid metabolites are not correlated with the size of the liver in sharks. The novelty in our study resides in ascertaining whether researchers can determine shark ‘health’ from simple overall metrics of body shape (body condition), which would provide significant insights into the greater ecology of large mobile predators. We discuss our results in the context of how condition and energy stores could impact critical phases of life history in sharks.

## Materials and methods

### Study species

Tiger sharks were sampled for this study in two subtropical locations: off the west end of Grand Bahama Island in the Bahamas (∼26.59°N, 79.08°W; ‘Tiger Beach’) in July 2012 and October 2013, and along a latitudinal gradient from Solider Key, Biscayne Bay, FL, USA (25.59°N, 80.16°W) to the reef edge in the middle Florida Keys in US federal waters (∼24.69°N, 80.85°W) from March to October 2013. All sharks were captured using standardized circle-hook drumlines (for specifics see Gallagher *et al.*, 2014). When a shark was captured, it was brought in swiftly (average time 1 min) and restrained and secured on a partly submerged platform on the boat. A water-pump was placed in the shark's mouth to permit irrigation of gills throughout sampling. While hooking durations ranged from 20–100 min for all individuals examined, it is unlikely that plasma metabolite values were influenced by an individual stress response to fishing, because tiger sharks have a dampened stress response to fishing (Gallagher *et al.*, 2014).

### Measurements and condition metrics

There is a rich literature on quantifying condition in fish (e.g. Cone, 1989; Stevenson and Woods, 2006; Pope and Kruse, 2007), including a very comprehensive analysis of condition in 2120 dead dusky sharks (*Carcharhinus obscurus*; Hussey *et al.*, 2009). These metrics typically involve either some relationship between length and mass or a direct measure of liver mass. Several reviews have discussed the advantages and pitfalls of such length–mass metrics of condition, and their remains debate as to whether they represent animal health accurately (Jakob *et al.*, 1996; Green, 2001). Given that we captured and released massive (>300 kg) tiger sharks, obtaining body masses or liver masses was not possible. While some studies have attempted to generate estimated body masses from sharks from various length–weight relationships (e.g. Mollet and Cailliet, 1996), it would not be appropriate for us to use masses calculated from length–weight models, using measured lengths and then dividing those estimates of mass by the same lengths that generated those masses to calculate intra-specific variation in shark condition. Such an approach would be inaccurate and circular. Rather, we opted to use a metric that employs independent measurements of body girth at different points along the body axis to estimate the overall body form of the shark (following Irschick and Hammerschlag, 2014; described below).We did, however, estimate the body mass of each shark (in kilograms) from the measured fork length (FL) by using the published length–weight relationship for tiger sharks presented by Kohler *et al.* (1995).

**Figure 1:**
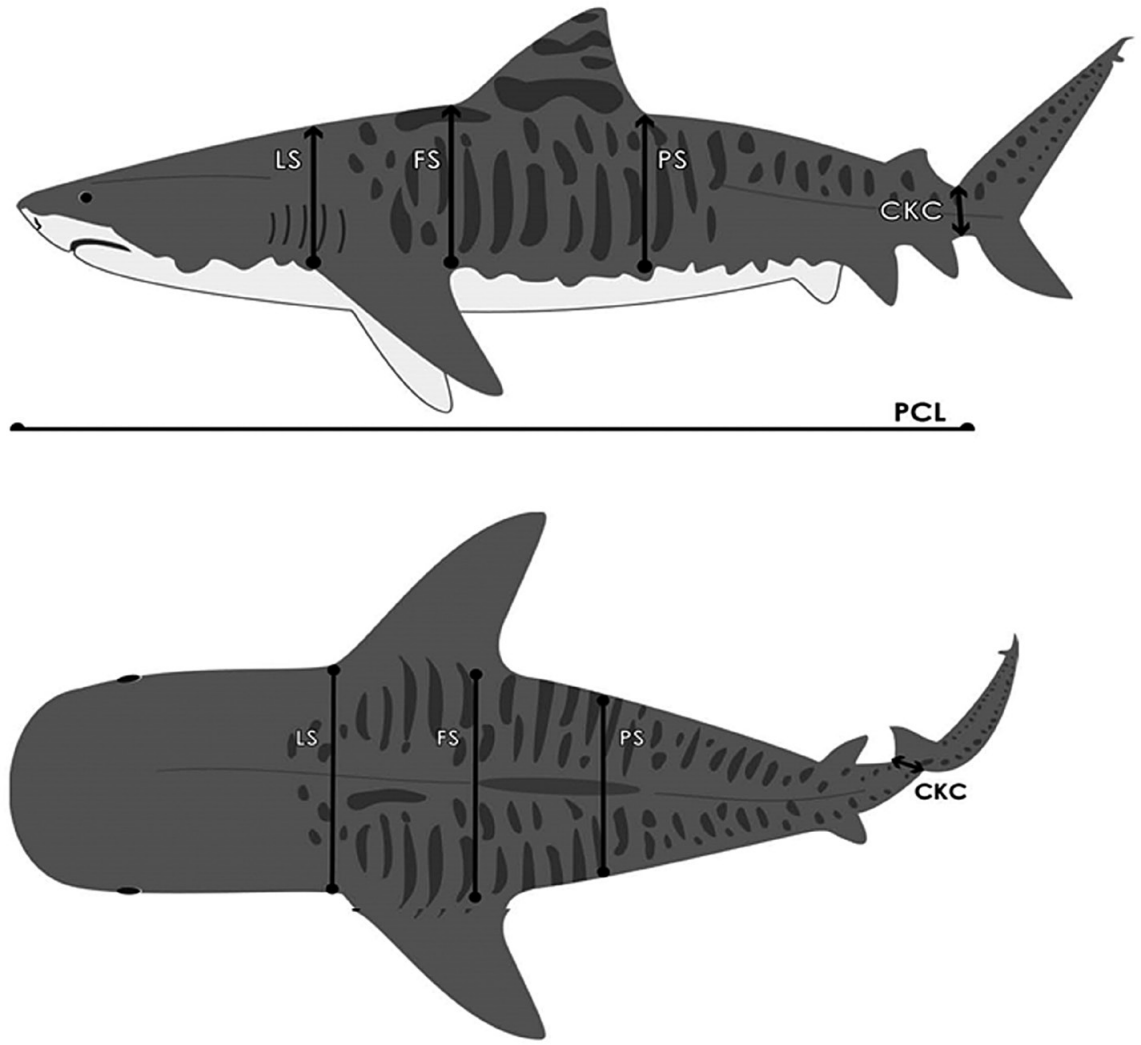
A diagram of a tiger shark identifying the morphological variables used in measuring condition. All four variables shown (CKC, caudal keel circumference; FS, frontal span; LS, lateral span; and PS, proximal span) were measured across the body.

While a simple measure of girth at any one point around the body can provide an index of size, sharks are not uniform in shape across their body, and their body proportions often vary among and between species. The index of body condition we employed was calculated by collecting several measurements of the body dimensions along the dorsal surfaces of each shark, as follows (Fig. 1): (i) pre-caudal length (PCL; linear distance from the tip of the snout to the insertion of the caudal fin into the body); (ii) lateral span (LS; distance between the insertion point of each pectoral fin; (iii) frontal span (FS; distance from the insertion point of the dorsal fin to a line emanating from the pectoral fin towards the tail); (iv) proximal span (PS; distance across the posterior edge of the dorsal fin as taken from the insertion point of the dorsal fin to a line emanating from the pectoral fin towards the tail); and (v) caudal keel circumference (CKC; circumference at the base of the tail around the pre-caudal pit). Condition (*C*) was defined as follows: *C* * = * Σ(LS + FS + PS + CKC)/(PCL). FL and total length (TL) were also measured.

### Blood collection and analysis

We collected 20 ml of mixed venous blood from the caudal vein of each shark using 18 gauge needles and 10 ml syringes. Approximately 7 ml of whole blood was aliquoted and centrifuged at 1300 ***g*** for 5 min to separate whole blood from plasma. Plasma was flash frozen in a dry shipper charged with liquid nitrogen. These samples were then transported to the Rosenstiel School of Marine and Atmospheric Science and stored at −80°C. A subset of plasma samples (six sharks from the July 2012 Bahamas trip and three Florida sharks) were frozen at <− 20°C before processing. We analysed plasma triglycerides (in millimoles per litre) using the EnzyChrom Triglyceride Assay Kit (ETGA-200; BioAssay Systems, Haywood, CA, USA; Liss *et al.*, 2013).

To ensure that there was no influence of freezing temperature on triglyceride values from samples frozen at different temperatures, we statistically compared the means from a set of paired samples frozen at −20 and −80°C from the Bahamas 2013 trip and found no significant differences (Student's paired *t* test assuming equal variances, *t* = −1.21, *n* = 11, *P* = 0.24); therefore, samples were pooled for analysis. Samples were run in triplicate across two plates with intra- and inter-assay coefficients of variation of 9.3 and 21.1%, respectively. The inter-assay variation is likely to be attributable to the very low (approaching zero in some cases) triglyceride values found in these animals. Data were normalized to meet the assumptions of normality. We evaluated the relationships between triglycerides and length, estimated weight and body condition, as well as the relationships between body condition and length and estimated body mass, using linear regression. Statistical significance was declared at *P* < 0.05, and all analyses were conducted using the R statistical program (R Development Core Team, 2009).

## Results

**Figure 2:**
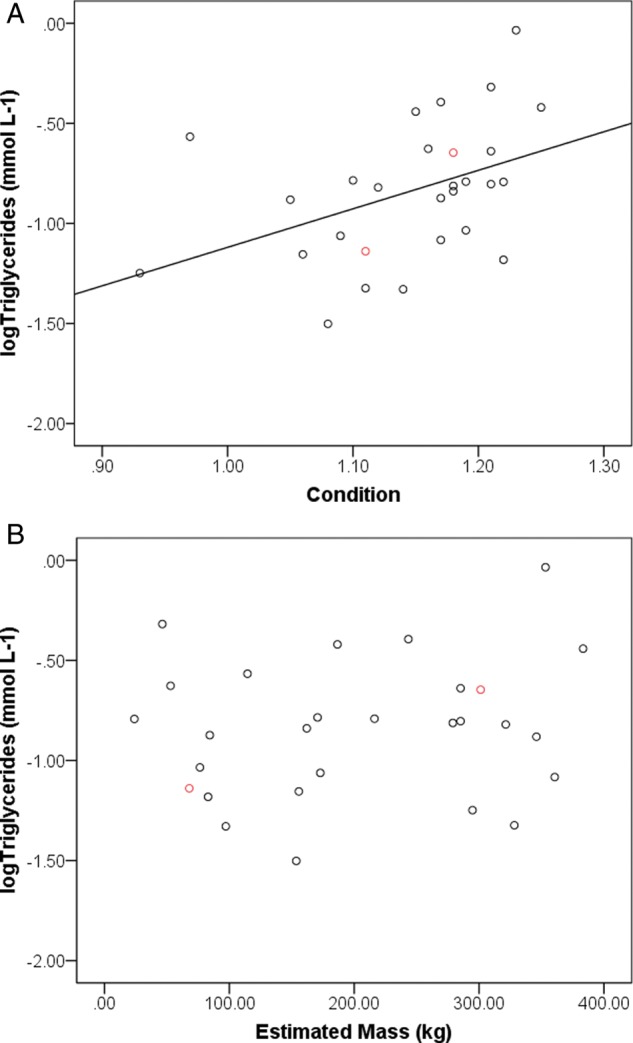
Relationships between plasma triglycerides (in mmol l^−1^) and condition (**A**), as well as weight (**B**; in kilograms, based on a published species-specific length–weight equation from Kohler *et al.*, 1995), in 28 free-ranging tiger sharks (black circles, females; and red circles, males) sampled in the subtropical Atlantic.

A total of 28 tiger sharks were sampled (26 females and two males), ranging in size from 125 to 303 cm (PCL, mean ± SD 230.54 ± 48.43 cm). Condition indices ranged from 0.93 to 1.25 (1.14 ± 0.08), with lower values generally indicating a ‘leaner’ shark. Sharks exhibited substantial variation in raw triglyceride values, ranging from 0.03 to 0.92 mmol l^−1^ (mean ± SD 0.20 ± 0.18 mmol l^−1^; Table 1). We detected a positive and significant relationship between body condition and triglycerides (*F*_1,27_ = 5.81, *P* < 0.05, *R*^2^ = 0.16; Fig. 2A). There were no significant relationships between triglycerides and length (PCL; *F*_1,27_ = 0.31, *P* = 0.58) nor between condition and length (*F*_1,27_ = 0.61, *P* = 0.44). Furthermore, there were no significant relationships between triglycerides and estimated body mass (*F*_1,27_ = 0.74, *P* = 0.40; Fig. 2B) nor between condition and estimated body mass (*F*_1,27_ = 0.20, *P* = 0.66).

**Table 1:** Biological, morphological, condition and triglyceride values for 28 tiger sharks sampled in the Bahamas and Florida in 2012 and 2013

Shark ID	Group	Sex	PCL (cm)	FL (cm)	TL (cm)	Estimated weight (kg)	Condition	Triglycerides (mmol l^−1^)
355	Bahamas 2012	F	226	245	296	155.67	1.06	0.07
111	Bahamas 2012	F	230	252	305	170.64	1.1	0.16
349	Bahamas 2012	M	172	190	232	67.95	1.11	0.07
341	Bahamas 2012	F	180	197	243	76.46	1.19	0.09
247	Bahamas 2012	F	182	202	248	82.97	1.22	0.07
136	Bahamas 2012	F	125	138	177	23.96	1.22	0.16
1222	Bahamas 2013	F	272	298	357	294.77	0.93	0.06
257	Bahamas 2013	F	200	223	273	114.54	0.97	0.27
1226	Bahamas 2013	F	290	313	380	345.95	1.05	0.13
1224	Bahamas 2013	F	222	244	292	153.60	1.08	0.03
1229	Bahamas 2013	F	225	253	305	172.86	1.09	0.09
1221	Bahamas 2013	F	281	308	368	328.26	1.11	0.05
1223	Bahamas 2013	F	278	306	357	321.36	1.12	0.15
1225	Bahamas 2013	F	190	212	260	97.13	1.14	0.05
215	Bahamas 2013	F	303	323	378	383.31	1.15	0.36
290	Bahamas 2013	F	248	281	331	243.39	1.17	0.40
222	Bahamas 2013	F	182	203	245	84.32	1.17	0.13
221	Bahamas 2013	F	281	317	373	360.58	1.17	0.08
1220	Bahamas 2013	M	269	300	356	301.27	1.18	0.23
1232	Bahamas 2013	F	266	293	353	278.95	1.18	0.15
246	Bahamas 2013	F	243	271	322	216.27	1.19	0.16
1219	Bahamas 2013	F	273	295	357	285.20	1.21	0.23
1227	Bahamas 2013	F	265	295	360	285.20	1.21	0.16
1228	Bahamas 2013	F	286	315	368	353.21	1.23	0.92
225	Bahamas 2013	F	233	259	307	186.58	1.25	0.38
327	Florida 2013	F	160	176	220	52.95	1.16	0.24
397	Florida 2013	F	224	248	289	161.97	1.18	0.14
427	Florida 2013	F	149	169	206	46.38	1.21	0.48

Abbreviations: F, female; FL, fork length; M, male; PCL, pre-caudal length; and TL, total length.

## Discussion

Animal condition has widely been viewed as a window into animal ‘health’, and thus, potentially impacts the ability of animals to feed, reproduce and migrate (Krebs and Singleton, 1993; Jakob *et al.*, 1996; Weatherhead and Brown, 1996; Green, 2001; Schulte-Hostedde *et al.*, 2001; Bearhop *et al.*, 2004; Goymann *et al.*, 2010). However, as mentioned earlier, common metrics of animal condition are not always simply related to independent metrics of physiological state. Therefore, understanding the relationships between animal condition and other metrics of energetic state is important for animals of conservation concern (Stevenson and Woods, 2006), especially for animals that migrate long distances and that require large energetic stores to reproduce (Liss *et al.*, 2013).

The vast majority of research on the energetic limitations of migration has occurred in birds. This work has revealed that individuals vary in individual condition and fuel reserves, which in turn influences their migration success, as well as the total fuel reserves available to birds once migration is completed (e.g. Merlla and Svensson, 1997; Bearhop *et al.*, 2004; Goymann *et al.*, 2010); however, findings for birds may not be applicable to other kinds of animals. Plasma metabolite concentrations of triglycerides have been correlated with higher rates of lipid catabolism and body mass (condition) before and during energetically demanding life-history phases in birds (e.g. Jenni-Eiermann and Jenni, 1992).

**Figure 3:**
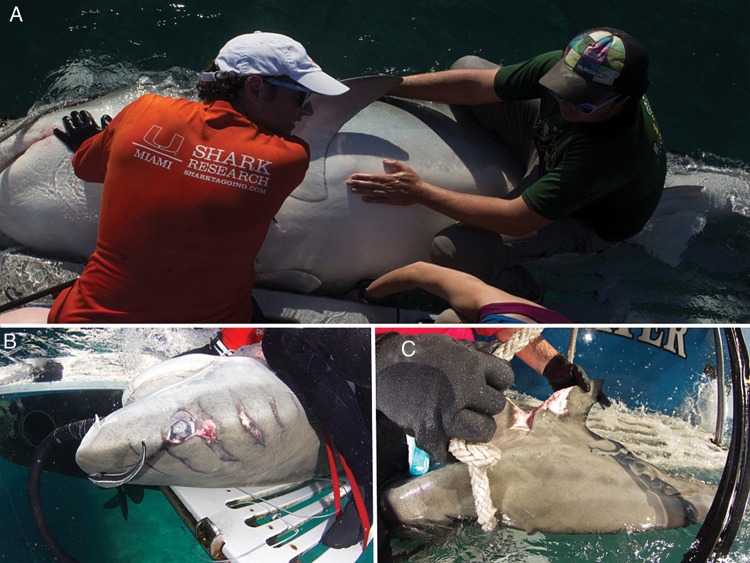
(**A**) An example of a high-condition/high-triglyceride value tiger shark with large girth from the present study, captured and sampled in October 2013. (**B** and **C**) An example of a low-condition/low-triglyceride individual captured on the same day, exhibiting physical trauma that is likely to have resulted from males biting the face and tail during mating.

We found that condition values predicted triglyceride values across a size range of tiger sharks. This finding is of general importance because it suggests that sharks with high ‘girth’ (i.e. fat sharks) also tend to possess larger energetic reserves (Fig. 3). This presents researchers with a simple metric for assessing health in sharks (condition) and will allow researchers an additional method to address questions concerning relationships between overall health, ecology and behaviour in top predators (Fig. 3). Our study did not, however, examine other aspects that are likely to impact condition values and triglyceride values. For example, in an analysis of 2120 dead dusky sharks (*Carcharhinus obscurus*), Hussey *et al.* (2009) showed seasonal changes in several metrics of condition. It would therefore be useful to examine seasonal changes in both triglyceride values and condition in tiger sharks, which would be especially valuable given the seasonal migrations that tiger sharks undergo and potential greater demands placed on female sharks during pregnancy.

The liver is the largest organ in most shark species and is likely to play an important role during lipid metabolism by driving a change in blood triglyceride levels during different phases of life (Oguri, 1990). During reproduction in sharks, the liver is involved in yolk production via vitellogenin production (Lucifora *et al.*, 2002). Moreover, female sharks store large amounts of lipids in the liver prior to the pre-vitellogenic phase of the reproductive cycle, to be consumed during vitellogenesis and gestation. Lucifora *et al.* (2002) found a negative correlation between liver weight and maximal diameter of ovarian follicles in sand-tiger sharks (*Carcharhinus taurus*). A recent study of white sharks (*Carcharadon carcharias*) satellite tagged in the Eastern Pacific found that sharks undergoing extensive migrations (4000 km) displayed a consistent increase in vertical drift rate, speculated to be due to a decrease in buoyancy caused by depleted lipid reserves during migration (Del Raye *et al.*, 2013). Our data suggest that measuring condition in white sharks may provide a stronger test of this hypothesis.

We noted a large amount of variation in both condition and triglyceride values in our sample of tiger sharks. Some of this variation may be related to differential use of liver energy reserves among individuals throughout movement phases and reproductive stages. While elasmobranch fishes seem to have, in general, much lower circulating levels of triglycerides than most other vertebrates, triglycerides have been well documented as a potential primary energy source, which often comprise a large proportion of the total lipid content in the liver (Ballantyne, 1997). At this point it remains unknown whether circulating plasma triglycerides are linked to either exogenous (external factors, such as recent ingestion) or endogenous processes (utilization within the body). However, the lack of high-fat prey at the Bahamas ecosystem (i.e. long time lags in high-energy resource input) and evidence from published data on tiger shark movement at the site likely suggest a within-individual variation of utilization of triglycerides which, in turn, may underscore a potential physiology/life-history trade-off between energy allocation to either soma or reproductive construction and maintenance costs (Zera and Harshman, 2001). While the observed variation among individual triglyceride values is likely to be a driving factor of a low *R*^2^ value (Fig. 2), this finding underscores the fact that life-history traits are often variable in natural populations and that negative associations (i.e. trade-offs) are generally a crucial assumption of life-history evolution.

In a study of the same population of tiger sharks at the Bahamas site (but none of the same individuals we sampled here), Hammerschlag *et al.* (2012) found that sharks exhibited a combination of two primary habitat use patterns, namely large-scale migrations (3500 km) with an activity space of 8549 km^2^ and high-residency patterns (>60 days) within 350 km^2^ of the tagging site. Furthermore, the majority of the individuals seen at the Bahamas site are sexually mature size classes and bear strong evidence of recent mating year-round, and especially in the spring (Hammerschlag *et al.*, 2012; A. J. Gallagher *et al.*, unpublished observations; Fig. 3B and C). It would therefore be valuable to compare condition and triglyceride values for these two groups of individuals and between sexes to determine whether energy accumulation during migrations precedes mating and gestation at high-residency sites. If this pattern is indeed occurring, tiger sharks may resemble ‘capital breeders’, in which provisioning of offspring is not limited by the travel time to and from foraging grounds (Costa, 1993). The movement data from Hammerschlag *et al.* (2012), documented generalist dietary patterns and low daily ration of this species (Gallagher *et al.*, 2011; Hammerschlag *et al.*, 2013) support this hypothesis. Future research expanding to include additional nutritional parameters (e.g. cholesterol and free fatty acids) and adding reproductive hormone assay, ultrasounds and movement data is needed to evaluate further these patterns and energy use in large elasmobranchs.

It is worth noting that, prior to normalization, raw triglyceride values were slightly left skewed (i.e. few individuals at the highest energy states). If this is pattern is truly representative, it could have important conservation implications. For example, if a high threshold level of energy stores is required for onset of pregnancy in tiger sharks, then their populations could be more vulnerable to anthropogenic threats that alter energetic intake.

In conclusion, our study highlights the potential role of physiology for organismal behaviour, fitness and demography (Cooke *et al.*, 2013; Cooke, 2014). Additionally, it underscores the utility of coupling investigations of body form with physiology to explore possible limitations on migration and other energetically challenging life-history phases in top marine predators using non-lethal techniques (Hammerschlag and Sulikowski, 2011). As researchers studying large predatory fish migration and behavioural ecology evolve to shift their attention from the ‘where’ to the ‘why’ and the ‘how’, studies using physiological measures of energy stores and condition may become increasingly valuable.
